# Current Impact, Future Prospects and Implications of Mobile Healthcare in India

**DOI:** 10.5195/cajgh.2014.116

**Published:** 2014-11-04

**Authors:** Rishi Kappal, Amit Mehndiratta, Prabu Anandaraj, Athanasios Tsanas

**Affiliations:** 1 MindActiv Consulting, Pune, India; 2 MIT School of Telecom Management, Pune, India; 3 Center for Biomedical Engineering, Indian Institute of Technology, Delhi, India; 4 Institute of Biomedical Engineering and Keble College, University of Oxford, United Kingdom; 5 School of Medical Science and Technology, Indian Institute of Technology, Kharagpur, India

**Keywords:** telemedicine, m-Health, telecommunication, mobile healthcare

## Abstract

India has a diverse geographical landscape and predominately rural population. Telemedicine is identified as one of the technological pillars to support healthcare services in this region, but is associated with high cost and complex infrastructure, thus restricting its wider use. Mobile-based healthcare (m-Health) services may provide a practical, promising alternative approach to support healthcare facilities. India has a high mobile user base, both in cities and in rural regions. The appropriate identification of mobile data transmission technology for healthcare services is vital to optimally use the available technology. In this article, we review current telecommunication systemsin India, specifically the evolving consensus on the need for CDMA (Code Division Multiple Access - a wireless technology used by leading international and national operators. This technology is deployed in 800MHz band making it economically viable and far reaching with high quality of services) to continue its operations in India along with mobile healthcare services. We also discuss how healthcare services may be extended using m-Health technologies, given the availability of telecommunications and related services.

## Introduction

India is the home to 1.24 billon people with a predominantely rural population. 1 Geographically, India’s landscape is diverse, with both mountainous and seafront regions. Furthermore, a large percentage of the population resides in villages who often have limited access to direct provision of primary healthcare services. Due to these challenges, the healthcare system is primarily managed by the government and complemented by various private healthcare institutes. Delivering medical services to remote areas is extremely intensive and often not viable for private healthcare providers.

While the government is primarily responsible for general healthcare, approximately 69% of the population has inadequate access to most health resources. 2 The number of public health facilities is also insufficient. For instance, India needs over 74,000 community health centers (CHC) per one million people, but it currently has approximately half that number. 3 Approximately 80% of total healthcare expenditure in the country is accounted for by those who see private healthcare providers; however, 70% of the healthcare infrastructure focuses almost exclusively on metropolitan cities. There is an impending need for innovation to maximize outreach and provide healthcare services to a wider population, primarily those in rural areas.

The Indian government has launched multiple projects to facilitate healthcare services in rural regions. 4 Telemedicine, one such project, may be able to facilitate the providing of vital healthcare services, even to the most remote parts of India. Telemedicine, by use of wireless technologies, will ensure that medical facilities are available to people in locations where physical healthcare facilities may be difficult to contruct, even in the most remote parts of India. Over the last ten years, the Indian government, along with private healthcare providers, has invested considerable research efforts and resources exploring the possibility of providing medical services and healthcare education to rural areas using telemedicine technology. 4,5

This article has three main foci of discussion. First, we address the current telecommunication environment in India, mobile technology, and services enabling m-Health. Second, we review published manuscripts, government reports, mobile healthcare provider’s web links, and publicly available articles on telecommunication and healthcare. The impact of telecommunication technology while considering the fate of m-Health in India is also highlighted. Third, we detail recommendations on which m-Health services can empower India at large.

## Telecommunication Services in India

In India, m-Health services are subject to regulators’approval based on governmental guidelines. These services are not owned by any particular industry, so the first step in setting standard operating procedures is done by the government. 6 The telecommunication industry (mobile and internet services) has grown exponentially in the last ten years, from under 37 million users in 2001 to over 846 million users in 2011. 7 India also has the world’s second-largest wireless phone user base, of which 37% are rural subscribers. 8 The urban states are hyper-dense with a tele-density (defined as the number of telecommunication facilities per one hundred individuals) of 154%, while rural India has a tele-density of only 40%. 8 Table 1 shows the rural and urban distribution of telecommunication services in India in 2012. 8

As of September 2012, approximately 50% of total wireless users have access to data services and the internet. 9 However, of the 446 million data enabled users that can potentially access the internet on their phone, only 79 million actually use the function. 9 There are many services available on mobile phones; however, not every mobile phone operator is informed of these services. While some operators activate data services by default with each connection, the gap between data ready phones and actively used data connections is wide due to the lack of education on the utility of such devices. This provides a sizeable window of opportunity to educate and connect rural India to receive m-Health services.

**Figure 1:**
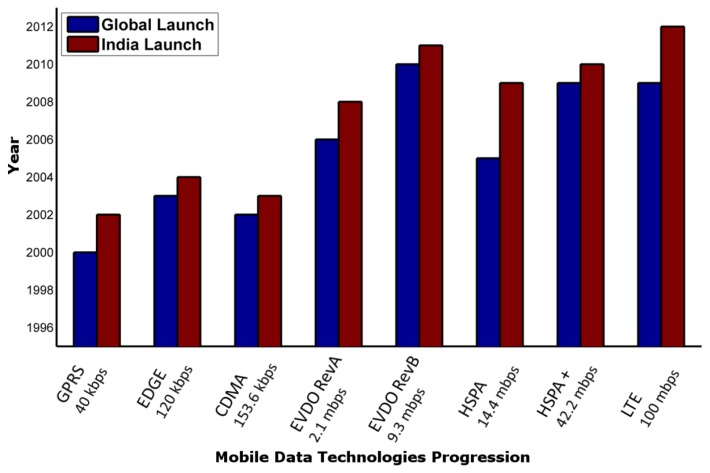
The trend of wireless technology (data speed) deployment in India and worldwide from 1996 to 2012.

Figure 1 depicts the status of wireless data technologies deployed in India, which gives the breadth of state-of-the-art telecommunications led data technology available for use. Wireless data technology deployment has lagged in India compared to global figures, but has succeeded by providing readily available, well-tested devices and services. In addition to existing broad- and narrow-band data technologies, facilitated by robust devices and services ecosystem, m-Health and telemedicine initiatives need only faster, economic services deployment and ownership by telecom ecosystem contributors in order to succeed.

Table 2 reflects the time lag of technology deployment and also maps the coverage of various wireless data technologies launched in India. General packet radio service (GPRS) and enhanced data for global evolution (EDGE) are narrowband technologies only available PAN India. The available wireless technology across the nation is code division multiple access (CDMA), enhanced by voice-data optimized (EVDO) RevA and RevB. This ecosystem of EVDO can be further enhanced to deliver last mile broadband connectivity, especially since the technology is deployed in the 850MHz spectrum. This provides the possibility of low CAPEX (Capital Expenditure) technology deployment and unmatched in-building coverage and penetration, making it viable for semi-urban and rural m-Health/telemedicine applications. Although these wireless technologies could help m-health services deploy in a more efficient manner, CDMA and EVDO technology subscribers have declined in India. The cost of acquiring patients and services for deployment can be adequately managed with CDMA/EVDO services and devices. Since multiple operators already have a passive infrastructure in each semi-urban town/village, the deployment of telecommunication is viable. This could save almost 30% CAPEX for operators and make m-Health services deployment more cost effective. 10

## Why Should Technology Matter for m-Health?

Stable and cost-effective technology for m-Health: CDMA, EVDO, GSM (GPRS and EDGE), and High Speed Packet Access (HSPA) are very stable technologies that are proven globally for various data transmission processes using mobile phones and other data access devices, like USB dongles.

CDMA, EVDO, and GSM (GPRS and EDGE) could be the most cost effective wireless technologies to be used in healthcare deprived areas of India. These technologies are more available, have the ability to connect most of the states, and incur very low cost to the end user (<$2 per month).

HSPA technology could provide a higher bandwidth for large data transmission by facilitating advanced healthcare services; however, it is not available in all areas of India and is expensive. The spectrum band of 2100MHz, in which HSPA is deployed in India, is a viable constraint, and available in only the 5MHz spectrum in 3G, a further bottleneck.

LTE TD (Long-Term Evolution, Time-Division Duplex) technology is still not deployed in India, particularly because most mobile devices are not equipped to support it. LTE TD rollout obligations are long standing and are offered in 2300MHz, making it CAPEX intensive (e.g., rollout of this technology is much more expensive as compared to rollout of wireless technology in lower frequency spectrums such as 700MHz and 800MHz frequency bands) and too far from reaching rural areas, leading to a low return on deployment.

Choosing the appropriate technology is an integral part for m-Health services to reach the wider population in India.

It is clear from the speeds and distances supported by these technologies that SMS based m-Health applications can be supported in all wireless frameworks. The choice of technology depends on the amount of data one needs to share between patient and doctor. Radiological images, vital statistics analyses, and single or multi-dimension data may require high data transmission protocols like EVDO and HSPA.

## m-HealthServices Available in India

In India, some of the mobile services providers have already initiated m-heath consultations. For example, Airtel (a leading mobile service provider in India) has formed a collaboration with Healthfore (a division of Religare Technologies) and Fortis Healthcare (a Religare group company) to offer Mediphone services to its mobile users. 11 By using this service, users are able to access basic medical guidance regarding non-emergency health problems over the phone. The service is available 24hrs a day for < $1 per consultation. 11 Other telecommunication service providers launched similar services in 2011 in collaboration with HealthNet Global, a Hyderabad-based emergency and healthcare management services firm. 12 Paramedical staff equipped with laptops, with high speed wireless technology, and medical diagnostic equipment consult with remotely located physicians using video conferencing and offer guidance to mobile phone users calling for healthcare advice. 12

In December 2011, Equitas micro finance also launched a tele-healthcare delivery center in association with HealthNet Global. 13 Equitas provides services such as “Consult 4 Health and Call 4 Health,” allowing its members to consult with Apollo Hospital physicians over video for approximately $1 per consultation. 13,14 The subject’s data may be stored for further assessment, treatment, and subsequent follow-ups. Other new models in m-Health are also emerging, like a Mumbai-based service called MeraDoctor (My Doctor) founded in 2010. 15 This service provides healthcare consultation over the phone to its members. The basic package of MeraDoctor costs approximately $17 per month per user (Rs 1000 pm). Another example is “3nethra” from a Bangalore-based Forus Health. 16,17 “3nethra” is a portable, non-invasive device, which could help in the early diagnosis of eye diseases such as cataract, diabetic retina, glaucoma and cornea related issues. 16,17 The digital information captured by “3nethra” can be easily transmitted electronically for analysis and consultation from experts at tertiary center hospitals located far from the screening center.

## India Landscape: Basic m-Health Using Wireless Technology

The most prolific and efficient technologies are between the spectrum band of 850MHz (CDMA) and 900MHz (GSM) for India. Some healthcare services that can be provided using this platform include:

1. SMS consulting: Increased access to mobile phones has led to the development of SMS services that connect users, allowing exchange of vital information and expert opinions in near real-time. This simple format allows users to ask time-sensitive questions anonymously to gain insight on potentially sensitive subjects. Concise information is easily spread, while being timely and conserving mobile battery.
2. E-education on healthcare matters: Mobile services can be used to broadcast useful healthcare-related information to a wide population. The development of faster mobile networks and improved device technology allows for an unprecedented level of quality content to be streamed and viewed. This enables access to the web’s entire library of video tutorials, how-to program, virtual classrooms, peer-to-peer chat on health tips, etc. Users can learn appropriateand home care techniques like first aid and up-to-date medical information.
3. Do it yourself (DIY) check-ups: It provides self screening tutorials like oral hygiene, insulin syringe injections, preparation of ORS (Oral Rehydration Solutions) for dehydration treatment, breast feeding techniques, or even self breast examinations for mass or tumours.
4. Improve drug compliance by mobile alert system: This program improves patient monitoring and drug compliance. One of the successful examples is multi drug therapy (MDT) for tuberculosis. This therapy must be administrated for 6 to 8 months and the failure rate for MDT is considerably high due to poor patient compliance. 18,19 Recent studies by Barcaly 18 and Kunawararak 19 *et al*. have shown that the treatment success rate could improved to over 90% by setting up an automated reminder to patients for drug either by SMS or a call.

## India Landscape: Advanced m-Health Using Wireless Narrowband and Broadband Technology

The EVDO Rev A and Rev B networks deployed by CDMA operators are the most efficient alternative for data intensive healthcare applications like real time patient consulting or Tele-radiology. Due to the lack of device ecosystem of CDMA in India, HSPA+ might be the next best alternative available. The potential use of EVDO and HSPA technology in facilitating healthcare services are elaborated in Table 3.

It is unclear as to which entities within the telecom ecosystem will take the lead in facilitating an m-Health solutionon the telecom network, encompassing medical experts for service delivery and making services reach the wider population in India, as well as determining billing mechanisms.

## Advanced m-Health Solutions for Chronic Diseases

In addition to tele-consultation, m-Health might be useful in providing more advanced services to patient in the form of diagnosis and monitoring of diseases. Research has demonstrated that mobile technology can provide the means for accurately monitoring symptom and severity for diverse pathologies, including diabetes, coronary artery disease, and Parkinson’s disease.

## Diabetic Monitoring

According to the International Diabetes Federation, India has the largest patient burden for diabetes mellitus, with approximately 50.8 million nationwide cases. 20 The prevalence of diabetes varies between 6 to 8% in the urban population and 2 to 3% in the rural population. 20 Mobile phone-based diabetic monitoring is currently in research and development. Many mobile applications have been developed to regularly record blood glucose in order to achieve a healthy lifestyle. 21,22 Patients can keep a record of their blood glucose on a mobile phone, and their levels can be sent to a physician through data protected mobile services. This simple, self-collected process may alleviate individuals from frequent physical visits to the clinic for glucose testing. The mobile application might also provide useful analysis tools based on previous blood glucose measures of the patient. Thus, a patient can now self-monitor his or her diabetes and plan an appointment with a physician only when required. This could be a very cost effective example of personalized mobile-based healthcare facility.

## Coronary Artery Disease and Remote ECG Monitoring

There are around 45 million patients in India with coronary artery disease (CAD); one-fifth of all deaths are caused by CAD. 20 At the current rate, it is expected that CAD will account for almost one-third of the total deaths by 2020. 20 In CAD management, it is important to regularly monitor the patient’s cardiac function, which involves regular visits with medical experts to perform an electrocardiography (ECG) examination. Regular ECGs can be a lifesaving procedure since it can indicate when patient might be at risk for myocardial infarction. Smartheart devices are designed to record the 12-lead ECG and can transmit securely encrypted data to a mobile phone. 23 The ECG signal may then be transmitted to a central processing unit at the patient’s hospital, where the medical team can assess and advise appropriate further action.

## Telemonitoring of Parkinson’s Disease Using Speech Signals

Parkinson’s disease (PD) is a neurodegenerative disorder affecting the central nervous system. Current prevalence of PD in India is approximately 3 million (diagnosed cases) and could be an additional 11 million undiagnosed cases according to a PD statistics report. 24 It is possible to record various trademark PD characteristics (such as limb tremor) via dedicated devices to remotely assess symptom severity. 25 However, many of these devices (e.g. accelerometers) are expensive and require careful placement to obtain reliable data. Recent research has shown the potential of using speech signals both to differentiate PD subjects from healthy controls 26 and also for telemonitoring PD symptom severity. 27 This aspect does not involve the use of specialized equipment, and the data is very easy to self-collect. Hence, someone using a mobile phone and a simple voice recording system could have direct access to an objective assessment of PD symptom severity. 28 Then, medical experts might provide guidance on optimizing pharmacological treatment.

## Potential for Growth

A recent report entitled “Global Telemedicine Market Analysis” by RNCOS Industry Research Solutions, 30 an India-based market research and information analysis company, projects that the global telemedicine market will grow at a compound annual growth rate (CAGR) of around 19% from 2010 to 2015. An earlier report in 2009, titled “Global Telemedicine Market: 2008–2012” published by Infiniti Research, 31 a London-based market intelligence firm, estimated the size of the global telemedicine market in 2008 at $9 billion. According to another report by the University of Pennsylvania, Asia is the fastest growing region for the telemedicine market, with India and China leading the growth. 29

The current size of the telemedicine market in India is difficult to be accurately estimated. Murali Rao, associate vice president for healthcare at the New Delhi–based research and consultancy firm Technopak Advisors, estimates the current size of the Indian telemedicine market to be around $7.5 million,^29^ suggesting “this is expected to grow at a [compound annual growth rate] of 20% over the next five years”, or approximately $18.7 million by 2017. Mehta of PwC on the other hand notes: “Studies indicate that the size of India’s telemedicine market is expected to be $500 million US by 2015.” 29

## Challenges Remain and Options Available

Various challenges still remain in the telemedicine market in India, despite the fact that telemedicine is gaining popularity around India.

### Choice of wireless technology

CDMA EVDO is a technology well-positioned to leverage m-Health and telemedicine solutions. However, with availability of other technologies, CDMA operators are returning precious spectrum back to government 32 and not contesting for more CDMA spectrum. This, in effect, will make CDMA dormant, and m-Health solutions on HSPA will not be as cost effective. Secondly, for wireless led healthcare, data networks should be stable and promise a ubiquitous experience across geographies. This, again, is compromised due to the overpriced and patchy HSPA spectrum presently and lack of investment continuity on HSPA from operators, since the existing investment awaits returns. Moreover, no operator has a PAN India HSPA presence. The available 2.3GHz LTE spectrum in India is a very CAPEX intensive setup to deploy. The LTE spectrum is on LTE TDD, which is less popular globally. LTE network owners restrict themselves to only the top 10 to 20 cities. In this scenario, it is important that a more cost effective wireless technology be launched in rural India. If the government allowed more CDMA/EVDO spectrum to be available to operators at an affordable price (in India, spectrum price is decided by the government), this would encourage operators to deploy a more affordable wireless technology to reach rural areas in India.

### Doctor-patient relationship

Mutual trust between patient and their doctor is important and is benefitted by the physical presence of both. In telemedicine or m-Health services, a virtual world might assist establishing a doctor-patient relationship. The potential of m-health in India is still under-realized because the lack of awareness among the population and lack of an appropriate service providing model. This would require an initiative from government agencies and private healthcare providers. In effect, m-Health services will not be successful until doctors are more enthusiastic and market m-Health as the most cost effective mode for providing quality healthcare in India. Well-recognized leaders in semi-urban and rural Indian states need to use telemedicine solutions. This will reduce perceived risk of relationships with other populations in the near vicinity. Also, projects like Gramjyoti 33 (launched by Ericsson India in collaboration with a large medical institute to demonstrate the benefits and use of 3G based telemedicine services in rural India) should be encouraged, in which the larger community sees delivery of a physical service through the virtual world.

### Strengthening of ecosystem and its awareness

The Indian telecom and medical ecosystem has limitations dependent on regulators, technology providers, technology deployment agencies, content providers, device developers, sales, and marketing companies. With so many factors, lack of ownership is imminent since each ecosystem partner will have to make sizeable investments. The telecomservice providers and patients need to be defined, facilitated, and executed. This, combined with technology and affordable devices, will be the tipping point. All parts of the telecom ecosystem must coexist to make a deployment solution mechanism.

### Standardization of solutions

In m-Health, the accountability of error needs to be examined when more service providers enter the domain. Standardization will lead to seamless adoption by relevant agencies and ensure timely upgrades so as to avoid redundant services. India’s partnering with global telecom standardization agencies like ITU-T and 3GPP/3GPP2 should develop telemedicine and healthcare service standardization authorities to lend a common approach to the partners at large.

### Reducing cost

The cost of m-health delivery still remains a challenge; however, it is expected that initiation of PAN India government funded projects might reduce the cost and increase the benefit of the technology. Also, by using CDMA as technology of choice, capital expenditure and operational expenditure optimization will lead to lesser cost of service delivery.

## Figures and Tables

**Table 1:** Total wireless users and tele-density in India with urban and rural distribution as of September 2012.

Total Wireless Users	906.62 million

Urban Wireless Users	571.70 million (63.06%)
Rural Wireless Users	33.49 million (36.94%)

**Table 2:** Technological details of current telecommunication services and its launch in India.

Data Technology	Data Speed	Global Launch Year	India Launch Year	India Coverage
GPRS 34	40 kbps	2000	Jan. 16, 2002 by BPL Mobile 35	Pan India by all GSM wireless operators in 900 MHz and 1800 MHz spectrum band

EDGE 36	120 – 384 kbps	2003	July 28, 2004 37,38	Pan India by all GSM wireless operators in 900 MHz and 1800 MHz spectrum band

1X CDMA2000 39	154 kbps	2002	May 2003 39	First launch by Reliance, followed by Tata Indicom, BSNL and MTS. Reliance and Tata provide Pan India 1X CDMA 2000 network, launched in 850 MHz spectrum band

EVDO Rev A 39	2.1 mbps upload and 1.8 mbps download	2006	2008 40,41	Launched by Tata under brand name of Tata Photon, followed by Reliance, BSNL and MTS in 850 MHz spectrum band. Tata 42 and Reliance 43 provide EVDO Rev A coverage Pan India

EVDO Rev B 39	9.3 mbps upload and 5.4 mbps download with 3 carriers	2010	Sept. 2011 44,45	Available in top 16 cities across India, by Tata, Reliance and MTS, in 850 MHz spectrum band

HSPA/HSPA+ 46	Up to 14.4 mbps/42 mbps with MIMO	2005/2009	Feb. 2009 limited launch by BSNL; 47 First private operator HSPA launch in India on Nov. 5, 2010, Tata Teleservices 48	No operator has Pan India 3G coverage on 2100 MHz spectrum band; 49 Limited 3G coverage and mobile broadband experience on HSPA+ yet to stabilize. Only 5 MHz of 3G spectrum allocated by government.
LTE 50	Up to 100 mbps	2009	April 2012 51	LTE-TDD in 2.3 GHz spectrum band, launched by Airtel and recently by Aircel. No further launches to date. Expensive technology with limitedLTE TDD handsets available.

**Table 3:** The potential use of EVDO and HSPA technology in facilitating healthcare services.

**Distance Learning and Diagnosis**	Mobile led video assisted healthcare awareness [20,21] Connecting to hospitals at a distance: In late 2009, hospitals in Sierra Leone launched a satellite link-up to connect doctors in the country to doctors in India where there is better equipment for data analysis. This facilitates real-time consultations between doctors in the field and specialists in hospitals Remote patient monitoring Web tutorials based healthcare [22] Web based anatomical display of symptoms and diagnosis [23] Self-help groups [24]
**Handheld Hospital**	Mobile phone based ophthalmic test equipment [25] Mobile phone enabled biometric and vital signs tester [26]
**Tracking Services**	Tracking medicines delivery [27] Smart labels for medicine time reminders [28] Mobile RFID based patients records tracking in hospitals [29]

## References

[1] (2012). India: World development indicators.

[2] Chandramouli C (2011). Census of India 2011: Rural urban distribution of population (provisional population totals).

[3] Park K (2009). Park’s textbook of preventive and social medicine.

[4] Mishra SK, Kapoor L, Singh IP (2009). Telemedicine in India: Current scenario and the future. Telemed J E Health.

[5] Ganapathy K (2002). Telemedicine and neurosciences in developing countries. Surg Neurol.

[6] Bedi BS (2009). Telemedicine standards: Need and Indian initiatives. Telemed J E Health.

[7] Telecom India http://www.imaginmor.com/telecom-india.

[8] Telecom Regulatory Authority of India (2012). Performance indicators report September 2012.

[9] Internet & Mobile Association of India Mobile internet in India.

[10] Association GSM (2012). Mobile infrastructure sharing.

[11] Airtel Mediphone.

[12] HealthNet Global HealthNet Global.

[13] Microfinance (2011). Equitas microfinance clients to get Tele Healthcare.

[14] India Infoline Apollo introduces Tele Medicine Centers in association with Healthnet Global.

[15] Meradoctor http://www.meradoctor.com/.

[16] Forus 3nethra.

[17] Global Commercialization Group 3nethra ophthalmology device.

[18] Barclay E (2009). Text messages could hasten tuberculosis drug compliance. The Lancet.

[19] Kunawararak P, Pongpanich S, Chantawong S (2011). Tuberculosis treatment with mobile-phone medication reminders in northern Thailand. Southeast Asian J Trop Med Public Health.

[20] New CarDiabCare http://neocardiabcare.com/alarming-statistics-india.htm.

[21] University of Florida Health Top-rated diabetes apps.

[22] Webicina Diabetes in social media.

[23] Telemedicine SHL (2014). Smartheart.

[24] Healthgrades Statistics by country for Parkinson’s Disease.

[25] Patel S, Lorincz K, Hughes R (2009). Monitoring motor fluctuations in patients with Parkinson’s disease using wearable sensors. IEEE Trans Inf Technol Biomed.

[26] Tsanas A, Little MA, McSharry PE, Spielman J, Ramig LO (2012). Novel speech signal processing algorithms for high-accuracy classification of Parkinson’s disease. IEEE Trans Inf Technol Biomed.

[27] Tsanas A, Little MA, McSharry PE, Ramig LO (2011). Nonlinear speech analysis algorithms mapped to a standard metric achieve clinically useful quantification of average Parkinson’s disease symptom severity. J R Soc Interface.

[28] Tsanas A (2013). Acoustic analysis toolkit for biomedical speech signal processing: concepts and algorithms.

[29] Knowledge @ Wharton University of Pennsylvania (2012). Can telemedicine alleviate India’s healthcare problems?.

[30] Market Research.com RNCOS.

[31] Infiniti Research Limited (2008). Global telemedicine market 2008–2012.

[32] The Economic Times (2013). Tata Teleservices to surrender excess spectrum in all circles barring Delhi, Mumbai 2013.

[33] Gram Jyoti http://gramjyoti.org.

[34] GSM Association GPRS (General Packet Radio Service).

[35] Cellular News http://www.india-cellular.com/News-Jan-Mar-2002.html.

[36] GSM Association Edge.

[37] Aditya Birla Group Idea.

[38] Prashant P (2004). Cellular service: Edge it’s got a long way to go.

[39] CDG CDMA History.

[40] Tata Photon Tata teleservices LTD.

[41] Tata Teleservices Tata group in communications.

[42] Tata Photon Tata Photon plus towns.

[43] Reliance Coverage.

[44] Tarun (2011). TTSL launches Photon Max REV B EVDO services on Tata DOCOMO.

[45] Bafna S (2011). MTS intros MBlaze ultra world’s first EVDO Rev B Phase 2 network.

[46] GSM Association HSPA.

[47] Cellular News BSNL launches 3G services in 11 cities.

[48] Light Reading India’s Tata ready for 3G launch.

[49] Broadband India Forum (2011). All India 3G network status + coverage.

[50] GSM Association LTE.

[51] LteWorld LTE Operators.

